# Grassland Degradation Changes the Complexity of Ant-Hemipteran-Plant Tritrophic Mutualisms

**DOI:** 10.3390/plants15121876

**Published:** 2026-06-17

**Authors:** Yuanyuan Feng, Yuxiao Zhang, Xiaoqian Yu, Meng Cui, Wesley Dáttilo, Yingzhi Gao

**Affiliations:** 1 Key Laboratory of Vegetation Ecology, Ministry of Education, State Environmental Protection, Key Laboratory of Wetland Ecology and Vegetation Restoration, Institute of Grassland Science, Northeast Normal University, Jilin Songnen Grassland Ecosystem National Observation and Research Station, Changchun 130024, China; fengyy766@nenu.edu.cn (Y.F.); zhangyuxiao101@nenu.edu.cn (Y.Z.); yuxq390@nenu.edu.cn (X.Y.); 2 College of Grassland Science, Inner Mongolia Agricultural University, Hohhot 010011, China; mcui@ibcas.ac.cn; 3 Red de Ecoetología, Instituto de Ecología AC, Xalapa CP 91073, Veracruz, Mexico; wesley.dattilo@inecol.mx; 4 Key Laboratory of Grassland Resources and Ecology of Western Arid Desert Area of the Ministry of Education, Key Laboratory of Grassland Resources and Ecology of Xinjiang Uygur Autonomous Region, College of Grassland Science, Xinjiang Agricultural University, Urumqi 830052, China

**Keywords:** grassland degradation, ant-hemipteran-plant, tritrophic mutualisms, mutualism mechanisms, root

## Abstract

Ants, plants, and hemipterans in tritrophic mutualisms represent closer approximations to real ecosystems compared to twofold mutualisms, playing a critical role in ecosystem functioning. Although habitat degradation is a useful framework for investigating the stability of mutualisms, few studies have focused on such mutualistic interactions in degraded grassland. In this study, we conducted both a field and a greenhouse experiment to assess the effect of grassland degradation on the organization of ant–plant networks and ant-hemipteran-plant tritrophic interactions in the light and severely degraded grassland of Songnen Plain, China. In general, we found that severe degradation of grassland changed the spatial distribution pattern of ant–plant networks from uniform to aggregation and increased the species diversity within these networks and facilitated the *Lasius flavus*-aphid/mealybugs-*Artemisia scoparia* tritrophic mutualisms. *L. flavus* improves individual plant performance by increasing plant height, reducing soil moisture content, and facilitating seed transportation of *A. scoparia*. These advantages enhance plant fitness and population spread of *A. scoparia*, consequently boosting its dominance within degraded grassland habitats. In turn, the well-developed root of *A. scoparia* attracted more *L. flavus* and aphid/mealybugs by providing living space and food. Our findings enhance the understanding of tritrophic mutualisms and their mechanisms in the context of grassland degradation, thus providing valuable information for the conservation, management, and restoration of degraded grassland.

## 1. Introduction

Grasslands encompass more than 30% of China’s overall land area and represent the largest terrestrial ecosystem in the country [1]. Since the late 1970s, extensive degradation has occurred in typical grasslands such as those on the Songnen Plain. Dominated by perennial *Leymus chinensis*, grasslands on the Songnen Plain constitute key pastures for grazing and hay cutting in China. Driven by growing human population pressure, overgrazing and agricultural reclamation have triggered severe degradation of native *L. chinensis* grasslands [2]. In addition, long-term aeolian sand deposition alters the composition of soil microbial communities, constraining soil carbon emission and carbon cycling and ultimately aggravating severe land degradation in this region [3]. The immediate consequence of this degradation is the emergence of extensive secondary bare saline-alkaline patches (BSAP) within the grassland, which creates environmental fragmentation and heterogeneity, further influencing soil fauna and flora in many ways [4]. This degradation not only alters the physical landscape but also can disrupt ecological processes (nutrient cycling, soil formation, seed dispersal, etc.), necessitating urgent measures for conservation and restoration to preserve the biodiversity and functionality of these critical ecosystems.

Ants (Hymenoptera: Formicidae) can shape the structure of vegetation communities, soil seed banks and soil properties in grassland as ecosystem engineers and have garnered widespread attention for their high resistance to grassland degradation [5,6]. For instance, there is empirical evidence that grassland degradation increases spatial heterogeneity, expanding the ecological niche space and allowing ants and plants to coexist and establish interaction networks [7]. In fact, ant-plant interaction relationships can be considered “key innovations” that drive species diversification by expanding niche breadth, geographic range, or population size, ultimately influencing speciation rates or reducing extinction rates [8,9,10,11,12]. This suggests that the complexity of such ant-plant relationships can play a pivotal role in determining community stability under the effects of habitat degradation. Moreover, the effect of environmental degradation on ants and plants involves a variety of interconnected mechanisms and processes operating at different spatial and temporal scales [13,14]. However, there is currently a lack of information on how the organization of ant–plant networks responds to grassland degradation, highlighting the need for further research on this topic.

In nature, there are various types of relationships between species, including mutualism, competition, predation, and parasitism. Among these, mutualism plays a crucial role in certain key ecosystems. The mutualistic symbioses of ant-plant and ant-aphid constitute typical representative cases. Ants act as key secondary seed dispersers and safeguard a wide range of plants against herbivore feeding. In turn, plants furnish ant colonies with food resources either directly or indirectly, alongside available nesting substrates [8]. Studies have found that ants such as *Lasius flavus* host abundant species of mutualistic root aphids inside their nest chambers [15]. Survival of several aphid species heavily relies on mutualistic partnerships with ants, which shield aphids from parasitoid wasps and predatory organisms. In return, ants acquire high-sugar honeydew frequently secreted copiously by aphids [16]. Third-party partners are commonly found in these mutualisms, often involving plant-based liquid resources (e.g., extrafloral and pericarpial nectar) [17], which represent closer approximations to real ecosystems than do twofold mutualisms. Hemipterans, lepidopterans and parasitoids have all been shown to be linked through trophic interactions with ants and plants [18,19,20,21]. The tritrophic mutualisms between ants and plants and honeydew-producing hemipterans have been most thoroughly investigated [22]. These tritrophic mutualisms usually involve plants providing resources (e.g., nesting space and food) to ants and hemipterans. In return, ants enhance plant adaptation by dispersing plant seeds and safeguarding plants from herbivores. Ants also regulate other invertebrates, including pest species, and protect other groups of insects such as aphids against parasites and predators. Hemipterans, such as the aphids and mealybugs, produce honeydew, which contains proteins, amino acids, sugars and other substances and provides important food resources for the ants [23,24,25,26,27,28,29]. Understanding these complex interactions is crucial for comprehending ecosystem dynamics and the roles different species play in maintaining biodiversity and ecological balance.

Currently, research on such tritrophic mutualisms has primarily focused on above-ground plant parts (e.g., domatia, extrafloral nectaries, and elaiosomes) [30], with less attention given to the role of plant roots. However, aphids, which serve as a source of food for ants, can also feed on plant roots [31]. Meanwhile, the presence of ants can substantially influence the biomass and morphology of roots through changes in the soil environment [32] and increasing arbuscular mycorrhizal colonization in grass roots [33]. Moreover, mycorrhized plant roots and the nest commensalisms (consisting of microbes and ants) absorb the available nutrients and increase biomass by lowering energy flows into runaway consumptions [34]. Therefore, it is necessary to include plant roots in studies of ant-hemipteran-plant tritrophic mutualisms to fully understand these complex interactions.

Mutualistic interactions are widespread and exhibit significant variability over time and across different locations [35]. In recent decades, understanding the biotic and abiotic factors driving these spatial and temporal variations has become a major focus in ecology and evolutionary biology [36,37]. Several studies have highlighted the importance of both biotic (such as species abundance, species identity, and plant phenology) [10,38,39,40] and abiotic (such as fire, temperature, and rainfall) [39,40,41] factors in shaping the dynamics of mutualisms involving ants, plants, and hemipterans. However, there is a scarcity of studies exploring the impacts of grassland degradation on mutualisms. Grassland degradation has major and long-term impacts on mutualisms, as the sustained presence of these species in the degraded grassland depends on the simultaneous existence of all partner species [42]. It has been demonstrated that the robust network structure formed by mutualistic species through their interactions with each other makes them more resistant to environmental disturbances. Nevertheless, such knowledge deficiency is further aggravated within tritrophic systems consisting of ants, host plants and sap-feeding Hemipteran insects; how grassland degradation reshapes their mutualistic network structures and interaction characteristics still remains poorly understood. Thus, we propose a hypothesis suggesting that the mutual influence among ant-hemipteran-plant engaged in mutualisms could play a crucial role in enhancing the survival and maintenance of each other’s communities in the degraded grassland. The intensive study of the ant-hemipteran-plant tritrophic mutualisms in the degraded grassland can promote the understanding of the processes and mechanisms of species tritrophic interactions to maintain ecological network stability when the environment is disturbed, and provides a framework for guiding the restoration of degraded grassland.

Our investigation began by examining the spatial distribution patterns, species diversity, and abundance of ants and plants in both lightly and severely degraded grassland of Songnen Plain, China. This area is notable for its lakes and wetlands, as well as its abundant water, biological, and land resources, making it one of the most biodiversity-rich regions [43]. Species abundance, diversity and heterogeneity of interactions are also important indicators of the complexity of mutualistic networks. So, the general objective was to understand how different degrees of grassland degradation impact the complexity of ant–plant networks. Subsequently, we further investigated the abundance of common plant species (*Setaria viridis*, *Leymus chinensis*, *Calamagrostis epigeios*, and *Artemisia scoparia*) on two dominant anthills (*Lasius alienus* and *Lasius flavus)* and surrounding vegetation to assess whether changes in plants are related to ants. Additionally, we quantified the number of ants and aphid/mealybugs on those common plant species to determine the relationship between ants, hemipterans, and plants. Furthermore, we analyzed the plant height, root morphology and nutrition of the common plant species, soil properties and soil seed bank, aiming to substantiate the underlying mechanisms of ant-hemipteran-plant tritrophic interactions in the degraded grassland. The significance of this study lies in its comprehensive exploration of the processes and mechanisms of ant-hemipteran-plant tritrophic interactions and their adaptation to the challenges posed by grassland degradation. Conservation strategies aimed at mitigating the impact of grassland degradation should carefully consider the intricate relationships between species, particularly those involved in tritrophic interactions, to enhance the resilience and sustainability of ecosystems in the face of environmental damage.

## 2. Materials and Methods

### 2.1. Study Sites and Experimental Design

The field study was conducted in 2015 at the Grassland Ecological Research Station of Northeast Normal University in the Songnen Plain (44°40′ N, 123°44′ E), a representative farming and pastoral area in northeast China. The aim of this study was to investigate the effects of degraded grassland on ant-hemipteran-plant trophic interactions. The region experiences an annual mean temperature ranging from 4.6 to 6.4 °C and annual precipitation between 300 and 500 mm, with 86% occurring during May to September. The soil in the area is alkaline, with a pH range of 8.5–9.5. In June 2015, we conducted a vegetation survey in the grassland, then divided it into two areas of light and severe degradation grassland. This classification was based on indicators that include the proportion of alkali spot area, vegetation coverage, above-ground biomass and plant species (Tables S1 and S2). Specifically, for lightly degraded grassland (LD), the average alkali spot area accounted for 18.5%, vegetation coverage reached 81.5%, and above-ground biomass was 588.8 g/m^2^; for severely degraded grassland (SD), the proportion of alkali spot area increased to 69.1%, whereas vegetation coverage and above-ground biomass declined to 30.9% and 520.6 g/m^2^, respectively (Tables S1 and S2). We established two experimental sites (light degradation and severe degradation, LD and SD) of 30 m × 100 m in the light and severe degradation grassland.

The greenhouse experiment was conducted in 2018 at the greenhouse of the Key Laboratory of Vegetation Ecology in Northeast Normal University. The greenhouse experiment aims to further understand the processes and mechanisms of species tritrophic interactions to maintain the stability of ecological networks. We selected *Artemisia scoparia* (Asteraceae), *Setaria viridis* (Poaceae)*,* and *Leymus chinensis* (Poaceae) to do the greenhouse experiment based on the results of the field vegetation survey, as they were the most abundant of all plant species (Table S3). Following that, we carefully transplanted the seedling-stage plants (5 cm in height) from the grassland into the greenhouse using specialized plastic bags. Immediately plant the seedlings in small trays and cultivate them in the greenhouse for 25 days. The default setting for the average minimum temperature in the greenhouse was 26 °C. The photoperiod was established at 16/8 h (Light/Dark), and humidity levels were maintained within the range of 50–70%. The experiment employed a completely randomized block design with five blocks, each block comprising three plant species (*A. scoparia*, *L. chinensis*, and *S. viridis*) and four treatments (plants without ants and aphid/mealybugs (CK), plants with only ants (L1AM0), plants with only aphid/mealybugs (L0AM1), and plants with both ants and aphid/mealybugs (L1AM1)). Once the plants had matured, carefully chose one plant per plant species and planted them in pots in the greenhouse according to the specified order (from left to right, *A. scoparia*, *L. chinensis*, *S. viridis*), 20 plants of each species in total. In accordance with the results of field experiments, we selected the *Lasius flavus* as the added ant species, with an introduction of 20 ants per pot. Additionally, 10 aphids (*Schizaphis graminum*) and 10 mealybugs (*Pseudococcus comstocki*) were added per pot. A white perforated mesh was employed to cover each pot, preventing the escape of ants and aphid/mealybugs. The two insect species (aphids and mealybugs) were selected mainly based on our field observational findings: individuals of these two insects were regularly distributed in clustered linear arrangements on the root systems of *A. scoparia* (Figure S3). This distinctive aggregation pattern inspired us to explore the underlying mechanisms driving such spatial distribution and their associated tritrophic interactions with *A. scoparia* and *L. flavus*, which constitute the core scientific rationale for selecting the two insect species in the present study.

### 2.2. Anthill Size and Spatial Distribution Pattern

In June 2015, we divided the 30 m × 100 m experimental site into 120 small 5 m × 5 m quadrats and conducted a statistical analysis of anthills within these small quadrats in the lightly degraded and severely degraded areas. The locations of all anthills were verified, and measurements were taken for the length of the long axis (D_1_, cm), minimum axis (D_2_, cm), and height (L, cm) of each anthill. Additionally, we quantified the anthill area (*S*, cm^2^) using the formula:

(1) $$S=\pi \times D_{1}/2\times D_{2}/2$$

and anthills mean crowding (m*) as:

m* = *m* + (*V*/*m* − 1) (2)

*m* is the mean density, and *V* is the sample variance. The distribution is random when m*/*m* = 1; the distribution is uniform when m*/*m* < 1; and the distribution is aggregated when m*/*m* > 1; *m* is the mean density of anthills [44].

### 2.3. Plant and Ant Characteristics

The Shannon–Wiener index for ants and plants was calculated by species richness and population abundance of ants and plants in the lightly degraded and severely degraded areas as:

(3) $$H=-\sum_{1}^{s}\left(P_{i}\times ln(P_{i})\right)$$

where *S* is the number of species; *P_i_* is the proportion of *i* among all individuals, *P_i_* = N*_i_*/N; N*_i_* is the number of individuals of *i*; N is the number of individuals of all species.

Furthermore, different from the selection of sample quadrats for the statistical analysis of anthills, we randomly selected 10 small 50 cm × 50 cm quadrats at the 30 m × 100 m experimental sites in the lightly degraded and severely degraded areas to record the abundance of common ant species (*Lasius alienus*, *Lasius flavus*, *Formica sinae*, *Tetramorium caespitum*) and the abundance of common plant species (*Setaria viridis*, *Leymus chinensis*, *Calamagrostis epigeios*, *Artemisia scoparia*).

### 2.4. Plant Characteristics of Anthills and Surrounding Vegetation

We further investigated the effect of ant dominant species (*L. alienus* and *L. flavus*) on plants. First, we selected 10 *Lasius alienus* anthills and 10 *Lasius flavus* anthills randomly as the sampling plots in the lightly degraded and severely degraded areas. Each control plot was separated from its respective anthill by 1 m (CK), thus forming a paired design. So, there were 2 degradation levels (lightly degraded and severely degraded), 2 anthill treatments (anthills and CK), 2 species (*L. alienus* and *L. flavus*), 10 repetitions, and in total, there were 80 sampling plots. Then we selected a quadrat of 50 cm × 50 cm randomly in each sampling plot in August 2015 and then recorded the abundance of dominant plants (*Leymus chinensis*, *Calamagrostis epigejos*, *Setaria viridis*, *Artemisia scoparia*) from the 80 experimental quadrats. Meanwhile, the height of each plant species was taken as the mean of 5 random plants (or fewer when 5 were not available) of the species.

### 2.5. Soil Properties and Soil Seed Bank

We also investigated the effects of dominant ant species *(L. alienus* and *L. flavus*) on soil properties. The selection of sampling plots was consistent with that used in the investigation of the effect of ant dominant species on plants. In late July 2015, soil samples (10 cm diameter) were collected to a depth of 10 cm from the 80 sampling plots. Three samples were collected at each plot and fully mixed, screened through a sifter of 2 mm after air drying, and then passed through a 0.15 mm sieve after stones and vegetation residue were removed manually. Soil pH and soil electrical conductivity were measured by the electrode method, soil moisture content by the oven drying method, and soil available phosphorus by the sodium bicarbonate leaching-molybdenum antimony colorimetric method.

Furthermore, we conducted an additional survey of the soil seed bank associated with anthills (*L. alienus* and *L. flavus*) and a control group (anthill-free blank sites selected at random). In October 2015, when ants ceased outdoor activity and after the plant seeds had fallen, we selected 10 anthills of *L. alienus*, 10 anthills of *L. flavus*, and 10 control groups in lightly degraded and severely degraded areas. Surface soil at a depth of 5 cm was collected using a cutting ring with a 10 cm diameter, dried under natural conditions, and subsequently cleared of insects, plants, root systems, and litter. The composition of the soil seed bank was determined through a Seed Germination Test (SGT). Seed germination was conducted in completely enclosed indoor chambers in 24 h cycles, comprising 12 h of light at 25 °C and 12 h of darkness at 20 °C. Seedlings were identified weekly, and those that were identifiable were removed until no new seedlings emerged.

### 2.6. Roots

In the field, six predominant plant species (*Setaria viridis*, *Leymus chinensis*, *Artemisia scoparia*, *Kalimeris integrifolia*, *Calamagrostis epigeios*, and *Artemisia anethifolia*) were chosen based on the vegetation survey, as they were the most abundant of all plant species (Table S3) conducted in July 2015. For each plant species, five anthills of *L. flavus* were designated as sampling sites, and control sampling sites were established 1 m outside the anthills (CK). The excavation area was delineated around the plant stem. We used shovels to excavate along the growth direction of roots to collect intact taproots, lateral roots and fine roots. Mechanical damage was avoided during the whole procedure to maximally maintain the original morphology of the root system. All samples were sealed in dedicated plastic bags to prevent aphids and mealybugs from escaping and then transported to the laboratory. First, the abundance of aphids and mealybugs on the roots was recorded. The roots were then rinsed with running water and preserved in plastic bags filled with 75% alcohol for laboratory analysis. Root morphology analysis (length, volume, surface area and tips) was performed using a scanner (Expression 1680 scanner, Epson Corporation, Suwa, Nagano Pref., Japan) and WinRhizo Pro 5.0 software. After root scanning, the samples were taken out. Surface moisture was gently blotted with absorbent paper, and then the samples were weighed using an electronic balance to obtain the fresh weight. Next, we studied the primary nutrient components of plant roots (the root moisture content, root crude protein contents, root acid detergent fiber and root soluble sugar content). The root moisture content (RMC) was obtained by subtracting the dry weight from the fresh weight. A ball mill (Mixer Mill MM 400, Retsch GmbH, Haan, Germany) machine was used to completely crush the roots for nutritional index measurement. Root crude protein contents (RCP) were measured according to the Kjeldahl method using Kjeltec 2300 automatic Kjeldahl apparatus (FOSS, Hillerød, Denmark); root acid detergent fiber (ADF) was determined by using Fibertec M6 fiber analysis system (FOSS Analytical A/S, Hillerød, Denmark) according to van der Waals (Van Soest) method, and root soluble sugar content (RSS) was calculated by anthrone colorimetric method. Finally, the samples were oven-dried at 65 °C to constant weight, and the root dry weight was then measured and recorded. The plants from the greenhouse experiment, which were collected approximately 45 days later, were promptly transported to the laboratory. The abundance of ants and aphid/mealybugs on the roots was recorded; later, root morphology and nutrient indexes were determined using the same method as described above.

### 2.7. Data Analysis

Normal distribution and homogeneity of variances were assessed for all data using Shapiro–Wilk tests conducted with SPSS 25.0 software (SPSS Inc., Chicago, IL, USA). One-way ANOVA was employed to investigate the effect of grassland degradation on the anthill’s height and area, the Shannon–Wiener index and the abundance of common ant and plant species. Additionally, two-way ANOVA was conducted to examine the effects of grassland degradation, anthills, and their interactions on the abundance and height of common plant species, soil properties, and the seed number of seed banks. Another two-way ANOVA was performed to investigate the effects of different plant species, the presence or absence of ants (aphid/mealybugs), and their interactions on the abundance of aphid/mealybugs (ants), as well as the effects of different plant species, the presence or absence of ants and aphid/mealybugs, and their interactions on the plant root morphology and nutrition. Differences among treatments were assessed using Duncan’s method for comparisons. Furthermore, we used the R statistical software v4.3.0 (R-Core-Team, 2024, Vienna, Austria) to analyze the biotic (ants and aphid/mealybugs) and abiotic (soil properties) factors that affect the abundance of *Artemisia scoparia*, and the relative importance of the effects of *A. scoparia*’s root indicator (root morphology and nutrition) on the abundance of *Lasius flavus* and aphid/mealybugs.

## 3. Results

### 3.1. Ant–Plant Networks Under Different Degrees of Grassland Degradation

**Figure 1:**
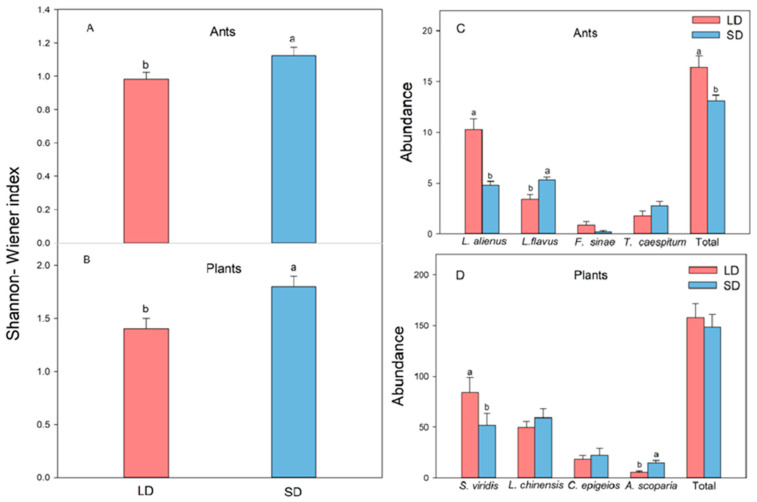
Shannon–Wiener index of ants (**A**) and plants (**B**) in the light and severe degradation sites. Abundance of common species of ants (**C**) and plants (**D**) in the light and severe degradation sites. LD: the light degradation sites; SD: the severe degradation sites. Different lowercase letters indicate a significant difference between LD and SD, *p <* 0.05. Values = means ± SE.

The coverage of vegetation was 81.5%, and the alkali spot area was 18.5% in the light grassland degradation sites (LD); the plants were evenly distributed. While the coverage of vegetation was only 30.9% and the alkali spot area was 69.1% in the severe grassland degradation sites (SD), the plants were distributed in aggregations (Table S1). In addition, the anthills in the lightly degraded area were mainly small anthills (S ≤ 0.4 m^2^), which occupied 88% of the total anthills. In contrast, the proportion of small anthills in the severely degraded area declined substantially (from 88% to 65%), while the proportion of medium anthills (0.4 m^2^ < S < 0.8 m^2^) increased (from 7% to 26%) (Table 1). Severely degraded reduced anthills intensity compared to lightly degraded, from 0.06 n/m^2^ to 0.042 n/m^2^. Moreover, the spatial distribution pattern of anthills in the lightly degraded area was uniform (m*/*m* = 0.867), while in the severely degraded area, the spatial distribution pattern of anthills was aggregated (m*/*m* = 1.392) (Table 1; Figure S1). Interestingly, grassland degradation increased the average area of anthills by 1.9 times in both the *Lasius alienus* and *Lasius flavus* (Table 1). Furthermore, severely degraded areas exhibited a greater area and height of anthills compared to the lightly degraded areas by 2.05 times and 1.29 times, respectively (Figure S2). The Shannon–Wiener index of both ants and plants in the severely degraded area was significantly higher than that in the lightly degraded area (Figure 1A,B). The total abundance of common ant species was significantly higher in the lightly degraded than in the severely degraded, especially for *L. alienus*, whereas the opposite was observed for *L. flavus* (Figure 1C). Regarding plants, severe degradation significantly reduced the abundance of *Setaria viridis* but increased the abundance of *Artemisia scoparia* (Figure 1D).

### 3.2. L. flavus-Aphid/Mealybugs-A. scoparia Tritrophic Mutualisms

**Figure 2:**
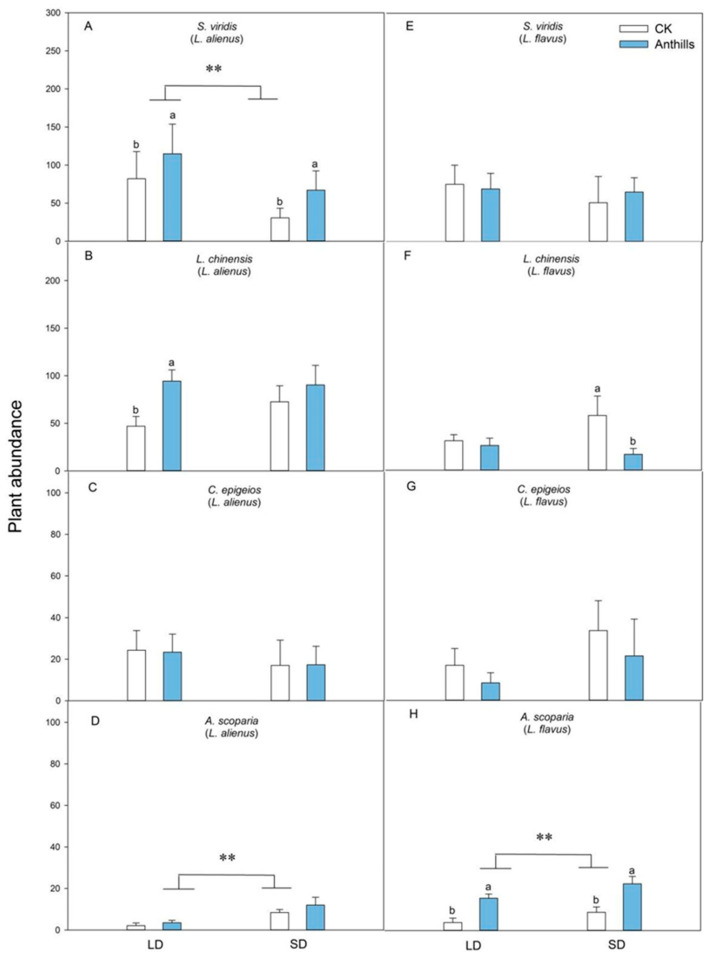
Effects of degradation and anthills on the abundance of different plant species. (**A**–**D**): *S. viridis*, *L. chinensis*, *C. epigeios* and *A. scoparia* on the *L. alienus* anthills; (**E**–**H**): *S. viridis*, *L. chinensis*, *C. epigeios* and *A. scoparia* on the *L. flavus* anthills. LD: the light degradation sites; SD: the severe degradation sites. Different lowercase letters indicate significant difference between anthills and surrounding vegetation (CK), *p* < 0.05; ** indicates significant difference between LD and SD, *p* < 0.01. Values = means ± SE.

We investigated the abundance of common plant species in the *L. alienus*, *L. flavus* anthills and surrounding vegetation (CK) to further verify whether ants influence the changes in plants under grassland degradation. As with our previous results, the abundance of *S. viridis* was indeed significantly reduced in the severely degraded areas compared to the lightly degraded areas on the *L. alienus* anthills (Figure 2A; Table S5), and *A. scoparia* significantly increased in the severely degraded areas compared to the lightly degraded areas, regardless of the type of anthills (Figure 2D,H; Table S5). Interestingly, compared to CK, *L. alienus* anthills significantly increased the abundance of *S. viridis* (Figure 2A; Table S5). In the lightly degraded areas, the abundance of *Leymus chinensis* in *L. alienus* anthills was also higher compared to the CK (Figure 2B; Table S5), but the opposite was observed on *L. flavus* anthills in the severely degraded areas (Figure 2F; Table S5). *L. flavus* anthills significantly increased the abundance of *A. scoparia* compared to the CK in the lightly degraded and severely degraded (Figure 2H; Table S5).

**Figure 3:**
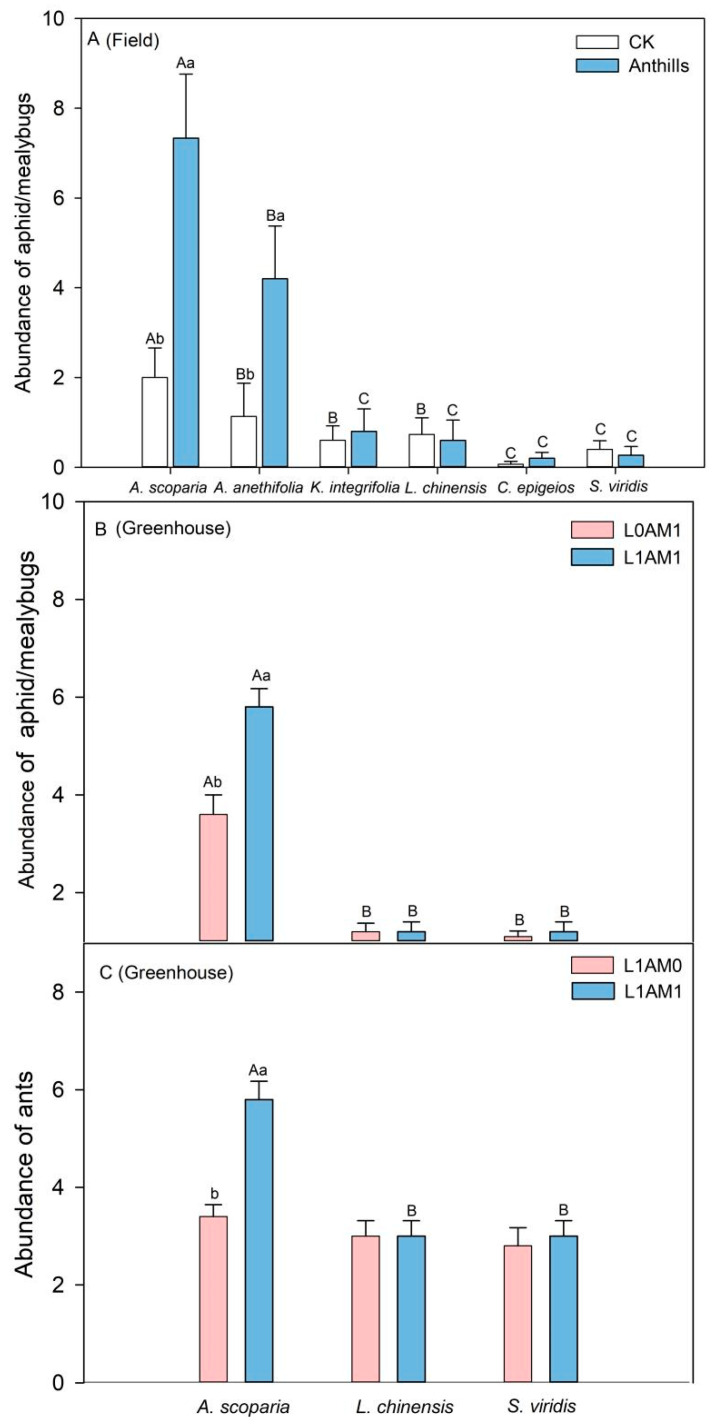
Abundance of aphid/mealybugs on the roots of different plant species in the field (**A**). Abundance of aphid/mealybugs (**B**) and ants (**C**) on the roots of different plant species in the greenhouse. Different capital letters indicate significant differences among different plant species; different lowercase letters indicate significant differences among different treatments, *p* < 0.05. Values = means ± SE.

Furthermore, our observations revealed that aphids and mealybugs dwelling on the roots of *A. scoparia* (Figure S3) establish a tripartite mutualistic symbiotic system with *A. scoparia* and *L. flavus*. Aphid/mealybugs abundance on plant roots was counted, and it was found that their abundance on the roots of *A. scoparia* and *A. anethifolia* was significantly influenced by the *L. flavus*, being significantly higher than that in the CK. Additionally, the abundance of aphid/mealybugs on *A. scoparia* root was significantly higher than that on *K. integrifolia*, *L. chinensis*, *C. epigejos*, and *S. viridis* root (Figure 3A; Table S6). Similarly, the results of the greenhouse experiment also showed that the presence of *L. flavus* increased the abundance of aphid/mealybugs, only on the root of *A. scoparia*; and the abundance of aphid/mealybugs on the root of *A. scoparia* was higher than on the other plant roots, regardless of the presence or absence of *L. flavus*. Interestingly, only on the root of *A. scoparia, L. flavus* abundance was significantly increased when the aphid/mealybugs were present; and the abundance of *L. flavus* on the root of *A. scoparia* was higher than on the other plant roots only when aphid/mealybugs were present (Figure 3B,C; Tables S6 and S7).

### 3.3. Mechanisms of Tritrophic Mutualisms

**Figure 4:**
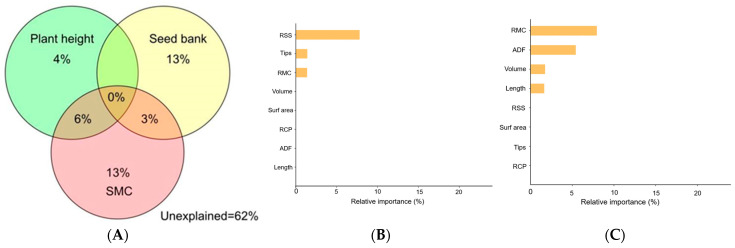
Venn diagram illustrating the unique and shared effects of the plant height, seed bank and SMC (soil moisture content) on variation in *Artemisia scoparia* abundance (**A**). Importance of different variables of root nutrition (RSS = root soluble sugar; RMC = root moisture content; ADF = acid detergent fiber; RCP = root crude protein) and root morphology (Surf = root surface area; Tips = root tips; Length = root length; Volume = root volume) to the ants’ abundance (**B**) and aphid/mealybugs abundance (**C**).

The Venn diagram illustrates the contribution of plant height, seed bank and SMC in explaining the variability of *Artemisia scoparia* abundance, which accounted for 4%, 13% and 13%, respectively (Figure 4A), and they are all affected by anthills. Firstly, the results for plant height showed that both *A. scoparia* and *S. viridis* growing in *L. alienus* and *L. flavus* anthills exhibited greater plant height compared to those growing in the CK, while anthills had no notable effect on the height of *L. chinensis* and *C. epigeios*. Furthermore, severe degradation significantly decreased the height of *A. scoparia* and *S. viridis* in the *L. alienus* anthills compared with lightly degraded, but the height of plants in *L. flavus* anthills was not affected by different degrees of grassland degradation (Figure S4; Table S8). Secondly, soil properties were similar in *L. flavus* anthills and *L. flavus* anthills. The soil of severely degraded areas exhibited relatively higher pH values and soil electrical conductivity, but relatively lower soil moisture content and soil available phosphorus than those of lightly degraded areas. In comparison to the surrounding soil, the anthills increased the soil available phosphorus but decreased the soil moisture content and soil pH of the anthill soil (Figure S5; Table S9). Finally, the quantity of *A. scoparia* seeds in the *L. flavus* anthills was significantly higher than that in the *L. alienus* anthills and the control group, irrespective of the degree of degradation. Meanwhile, the total seed quantity was similar between *L. flavus* anthills and *L. alienus* anthills but much higher than the control group (Table S4).

Random forest modeling results showed that root soluble sugar content (RSS), the number of root tips (Tips) and root moisture content (RMC) of *A. scoparia* effected *L. flavus* abundance (in descending order of relative importance) (Figure 4B); whereas root moisture content (RMC), root ADF content (ADF), root volume (Volume) and root length (Length) of *A. scoparia* effected aphid/mealybugs abundance (in descending order of relative importance) (Figure 4C). In the field, root morphology (including the root length, volume, surface area, number of tips and the length of different root diameters) of *A. scoparia* roots that grew in the *L. flavus* anthills was significantly greater than that in the CK and was significantly greater than that of other plants (Figures S6A–D and S7A; Table S10). The same results were found in the greenhouse experiment; the root morphology (including the root length, volume, surface area, number of tips and the length of different root diameters) of *A. scoparia* also exhibited a significant increase in the presence of *L. flavus* and aphid/mealybugs and was significantly greater than that of other plant species (Figures S6E–H and S7B; Table S10). Regarding the root nutrition, the RMC, RSS and ADF of *A. scoparia* root were also significantly higher than those of other plant roots. Meanwhile, the RMC and ADF of *A. scoparia* root were significantly reduced in the presence of *L. flavus*, and RSS and ADF of *A. scoparia* root were significantly reduced in the presence of aphid/mealybugs (Figure S8; Table S11).

## 4. Discussion

### 4.1. Degradation-Driven Spatial Redistribution and Diversity Responses

The dynamics and spatial distribution patterns of species populations, including population size, density, distribution and structure, are crucial in species population ecology [45]. Our results indicate that severe grassland degradation synergistically altered the spatial distribution of both ants and plants, shifting from a uniform pattern to an aggregation pattern (Table 1 and Table S1; Figure S1). Intraspecific competition and resource competition are crucial factors influencing the formation of species' spatial distribution patterns [46,47,48]. When the grassland was lightly degraded, resources were relatively abundant, leading to less intense individual competition for resources. However, intraspecific competition limited the quantity of ants and plants within a unit area, and individuals migrated to ensure each had enough space (typically of similar size) to survive [49], resulting in a uniform distribution pattern. This was supported by the evidence of small areas but high density of anthills under the light degradation sites (Table 1; Figure S1A). However, severe grassland degradation also led to the creation of large amounts of secondary bare saline-alkaline patches (BSAP) (Table S1), significantly reducing the availability of resources. Moreover, the alternating distribution of alkaline patches and “grass islands”, along with the nesting behavior of ants, introduced heterogeneity in the distribution of resources [50]. According to the spatially explicit habitat selection models of intuition, habitat selection for individuals living in heterogeneous landscapes primarily depends on the location of habitat patches and available resources [51]. The high concentrations of ants and plants in resource-rich areas, and the opposite trend in nutrient-poor areas, resulted in an aggregated distribution pattern, as evidenced by a large area but low density of anthills under the severely degraded sites (Table 1; Figure S1B). Additionally, the aggregated distribution pattern offered mutual shelter and access to favorable resources within clusters, enhancing survival and competitiveness, particularly in the degraded grassland, and contributing to the stability of the population itself [52].

Studies have recognized habitat fragmentation as a significant contributor to biodiversity loss [53]. Nevertheless, recent research has raised doubts about whether fragmentation invariably leads to species decline [54]. In this sense, our observations suggest that severe grassland degradation notably enhanced the diversity of ants and plants (Figure 1A,B). The reason may be attributed to the formation of heterogeneous landscapes resulting from severe grassland degradation, with ant diversity showing a tendency to increase in habitats with greater structural complexity [55]. Meanwhile, ants can enhance plant diversity through the transportation of plant seeds and/or the creation of favorable conditions for the establishment of plant communities [56].

### 4.2. Degradation-Driven Community Composition Reassembly and Stress-Tolerant Species Dominance

Indeed, the composition of species communities was discovered to be more sensitive to environmental changes [57]. The reason is that grassland degradation affects individual species differently; there is often a shift in the species composition, favoring the dominance of certain stress-resistant species [58]. In our study, the community composition of both ants and plants underwent changes under severe degradation, marked by a significant decrease in the abundance of *Lasius alienus* and *Setaria viridis*, and a notable increase in the abundance of *Lasius flavus* and *Artemisia scoparia* (Figure 1C,D). We speculate that the reason for the dominance of *L. flavus* under severely degraded grassland is that it is much smaller than other ant species, so very small patches could be enough to host viable populations [54]. Furthermore, *L. flavus* and *A. scoparia* mutually promote each other’s abundance, especially when mediated by aphid/mealybugs (Figure 2 and Figure 3), forming a special tritrophic mutualism, thus improving community stability in the degraded grassland.

### 4.3. Multi-Pathway Stabilization Mechanisms of the Lasius flavus-Aphid/Mealybug-Artemisia scoparia Tritrophic Interaction

**Figure 5:**
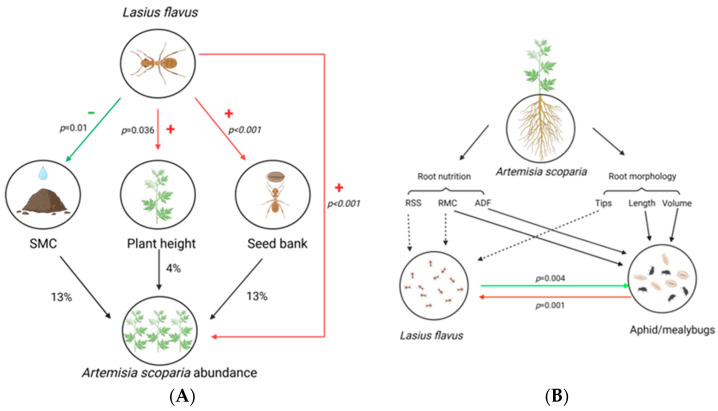
Mechanisms by which the *Lasius flavus* partially facilitated the establishment of *Artemisia scoparia* colonies (**A**). Green arrows represent attenuating effects of *Lasius flavus*, red arrows represent facilitating effects of *Lasius flavus*, and black arrows represent environmental factors affecting *Artemisia scoparia* abundance. Mechanisms of multi-trophic mutualism among *Artemisia scoparia*, *Lasius flavus* and aphid/mealybugs (**B**). Black dotted arrows indicate the effect of the *Artemisia scoparia* root on the *Lasius flavus* abundance; black solid arrows indicate the effect of the *Artemisia scoparia* root on the aphid/mealybugs abundance; green solid arrows indicate the effect of *Lasius flavus* abundance on the aphid/mealybugs; red solid arrows indicate opposite effects. The plus sign (+) represents positive effects, while the minus sign (−) indicates negative effects.

We further found the mechanisms of the *L. flavus*-aphid/mealybugs-*A. scoparia* mutualisms under degraded grassland as below (Figure 5): *L. flavus* may have partially facilitated the establishment of *A. scoparia* communities under severe grassland degradation through three pathways (Figure 5A). Firstly, *L. flavus* anthills significantly enhanced the plant height of *A. scoparia* (Figure S4), which explained the variability of *A. scoparia* abundance accounted for 4%. Grubb observed that the piling-up effect of anthills on the soil leads to the disappearance of most short weeds [59]. In our study, we found that severe grassland degradation further increased the height of the anthills (Figure S2). Therefore, the height-increasing effect of *L. flavus* anthills on *A. scoparia* provides it with a competitive advantage in the severely degraded grassland. Secondly, *L. flavus* can influence plant species composition and distribution by modifying the soil environment as ecosystem engineers [60,61]. The lower soil moisture content in anthill soil (Figure S5) creates a relatively dry environment suitable for *A. scoparia*, which grows well in temperate dry or moderately dry habitats [62]. Soil moisture content explained the variability of *A. scoparia* abundance, accounting for 13%. The final way was the preference of *L. flavus* to carry *A. scoparia* seeds (Table S4), which explained the variability of *A. scoparia* abundance, accounting for 13%. The reason is that dispersules’ weight limits seeds spreading from surrounding grassland plants to anthills [63]. There is research showing that the hundred-grain weight of *A. scoparia* seeds is only 5.17 g, which is far smaller than the weight of *Gramineous* plants’ seeds, and smaller seeds may possess more persistent seed banks [64]. Beyond the three pathways mentioned above, *L. flavus* may also indirectly promote the establishment of *A. scoparia* communities by modifying soil microenvironments. The burrowing and nesting activities of ants alter soil structure and improve soil aeration, which is conducive to root respiration and plant growth [65]. Meanwhile, changes in soil physical properties further reshape the composition of soil microbial communities and effectively suppress the abundance of plant pathogens, reducing the risk of disease stress for *A. scoparia* [66]. In addition, ants regulate the height of anthills, which changes the distance of solar radiation reaching the ground surface. This micro-topographic variation modulates the local microclimate and maintains a suitable temperature environment for plant survival and development [67]. These comprehensive effects jointly constitute important potential mechanisms driving the colonization of *A. scoparia* and also account for a large part of the unexplained variation in our statistical analysis.

From the plant’s perspective, they have developed a variety of strategies to compete for the partnership of ants and aphid/mealybugs. For example, some plant species can directly offer carbohydrates to ants through nectar from extrafloral nectaries (EFN) [68]. Such investments are often costly [69], so plants may lack sufficient resources to develop structures like EFN under degraded grassland. However, *A. scoparia* can develop stronger and more nutritious roots than other plant species under severe grassland degradation (Figures S6–S8). The *L*. *flavus* is an omnivorous ant species. Its food resources and energy required for nest construction are highly dependent on carbohydrates. Ants can directly utilize soluble sugars secreted by plant roots as an energy source [70]. Soluble sugars accumulated in the roots of *A*. *scoparia* serve as the primary energy supply for *L. flavus*. Higher sugar content creates stronger food attractiveness to the ants, sustaining the survival and activities of larger colonies. Accordingly, soluble sugar content in roots acts as the primary key factor driving the abundance of *L. flavus* populations. Root tips represent the most active region for plant metabolism and exudate release, continuously secreting carbohydrates, amino acids and other substances [71]. Abundant root tips further improve food supply and habitat conditions, constituting a secondary influencing factor. In addition, we found that the key root traits of *A. scoparia* regulating the abundance of *L. flavus*, aphids and mealybugs differ substantially. This discrepancy essentially stems from divergences in feeding preferences, survival requirements and ecological niches between the two groups of insects, as well as the distinct functional division within the tripartite mutualistic symbiosis. Unlike ants, root water content is the dominant factor affecting the abundance of aphids and mealybugs, while root volume and root length are the major morphological determinants. Aphids and mealybugs are hemipteran piercing-sucking insects that obtain nutrients by feeding on plant phloem sap throughout their lifespan [72]. Plant roots function as the core organ for water absorption and translocation, and root water content directly determines the volume and fluidity of phloem sap [73]. Sufficient water facilitates feeding and accelerates reproduction of piercing-sucking insects, whereas water deficit restricts their population expansion. Therefore, root water content is the leading factor influencing aphid and mealybug populations. Root length and root volume govern the distribution range, biomass and available feeding space of roots. More developed roots provide more feeding sites and support larger insect populations. Nevertheless, these morphological traits merely serve as spatial carriers and do not directly determine food quality, thus being less influential than water content and fibrous substances. In grassland ecosystems undergoing degradation with limited resources, *A. scoparia* does not selectively enhance a single root trait. Instead, it synchronously optimizes multiple root characteristics, including sugar content, water content, morphology and fibrous substances. Through such trait combinations, the plant accommodates the survival demands of ants and hemipteran insects, respectively, alleviating resource competition between the two insect groups and maintaining the stability of the tripartite symbiotic system. This represents an efficient adaptive strategy for symbiosis evolved by plants under stressful habitats.

## 5. Conclusions

Severe degradation of grassland has resulted in the formation of aggregated spatial distribution patterns and increased species diversity within ant–plant networks. Interestingly, a tritrophic mutualism involving *L. flavus* and *A. scoparia* and aphid/mealybugs becomes more prominent in the severely degraded grasslands. The mechanisms underlying this mutualism included: (a) *L. flavus* promoting the establishment of *A. scoparia* communities by enhancing plant height, decreasing soil moisture content, and facilitating the transportation of *A. scoparia* seeds; (b) the well-developed root of *A. scoparia* offered shelter and food for *L. flavus* and aphid/mealybugs. These findings advance the understanding of complex interspecific associations under environmental disturbance and demonstrate that tritrophic mutualism enhances population resilience and persistence within degraded grassland ecosystems. The results offer theoretical references for developing grassland conservation and restoration strategies, including selecting *A. scoparia* as a pioneer constructive species for the restoration of degraded saline-alkali grasslands via artificial seed supplementation to support population establishment, as well as conserving indigenous *L*. *flavus* populations and prohibiting pesticide application.

## Figures and Tables

**Table 1:** Distribution of anthills in the light and severe degradation sites. LD: the light degradation sites; SD: the severe degradation sites; S: anthills area; m*/*m*: the patchiness indicator of Lioyd [44].

	LD	SD
Small anthills (S: ≤0.4 m^2^)	157 (88%)	82 (65%)
Medium anthills (S: 0.4–0.8 m^2^)	12 (7%)	33 (26%)
Large anthills (S: ≥0.8 m^2^)	10 (5%)	11 (9%)
Anthills density (n/m^2^)	0.06	0.042
Anthills distribution type	m*/*m* = 0.867 (Uniform)	m*/*m* = 1.392 (Aggregate)
*L. alienus* anthills average area (m^2^)	0.211	0.409
*L. flavus* anthills average area (m^2^)	0.296	0.572

## Data Availability

The datasets generated during and/or analyzed during the current study are available from the corresponding author on reasonable request.

## Supplementary Materials

The following supporting information can be downloaded at: https://www.mdpi.com/article/10.3390/plants15121876/s1, Figure S1: Location and area of anthills in the light degradation sites and the severe degradation sites; Figure S2: Effects of degradation on the anthills height and area; Figure S3: Aphids (*Schizaphis graminum*) and mealybugs (*Pseudococcus comstocki*) on the plant roots; Figure S4: Effects of degradation and anthills on the height of different plant species; Figure S5: Effects of degradation and anthills on soil properties; Figure S6: The root morphology including length, volume, surface area and tips of different plants in the field; Figure S7: The root length of different root diameters of different plants in the field and greenhouse; Figure S8: The root nutrition including root moisture content, root soluble sugar, root ADF and root crude protein of different plants in the field; Table S1: Grassland degradation indicator in the light and severe degradation sites; Table S2: Description of plant community composition in the experimental sites; Table S3: Description of plant species abundance in the experimental sites; Table S4: Species composition and abundance of seed bank in the soil of anthills and surrounding; Table S5: Two-way ANOVA for the effect of degradation, anthills and interaction on the plant abundance of *L. chinensis*, *C. epigeios*, *A. scoparia* and *S. viridis* on the *L. alienus* and *L. flavus* anthills; Table S6: Two-way ANOVA for the effect of plant species, treatment and interaction on the abundance of aphid/mealybugs in the field, the abundance of aphid/mealybugs and ants in the greenhouse; Table S7: One-way ANOVA for the effect of ants abundance to the abundance of aphid/mealybugs in the root of *A. scoparia*, *L. chinensis* and *S. viridis*; and the effect of aphid/mealybugs abundance to the abundance of ants in the root of *A. scoparia*, *L. chinensis* and *S. viridis*; Table S8: Two-way ANOVA for the effect of degradation, anthills and interaction on the plant height of *L. chinensis*, *C. epigeios*, *A. scoparia* and *S. viridis* on the *L. alienus* and *L. flavus* anthills; Table S9: Two-way ANOVA for the effect of degradation, anthills and interaction on the soil moisture content, soil pH, soil electrical conductivity and soil available phosphorous of the *L. alienus* and *L. flavus* anthills soil; Table S10: Two-way ANOVA for the effect of plant species, treatments and interaction on the root morphology (root length, volume, length/volume, project area, surface area, tips, length of different root diameter); Table S11: Two-way ANOVA for the effect of plant species, treatments and interaction on the root nutrition (root moisture content, root soluble sugar, root ADF, root crude protein).
