# Supplementary material for: Grassland Degradation Changes the Complexity of Ant-Hemipteran-Plant Tritrophic Mutualisms
Source: Plants (Basel). 2026 Jun 17;15(12):1876. doi: 10.3390/plants15121876 (PMC13306811; doi:10.3390/plants15121876)
Supplement: Supplementary file 1 [file plants-15-01876-s001.zip › plants-4349953-Supplementary Material.pdf]

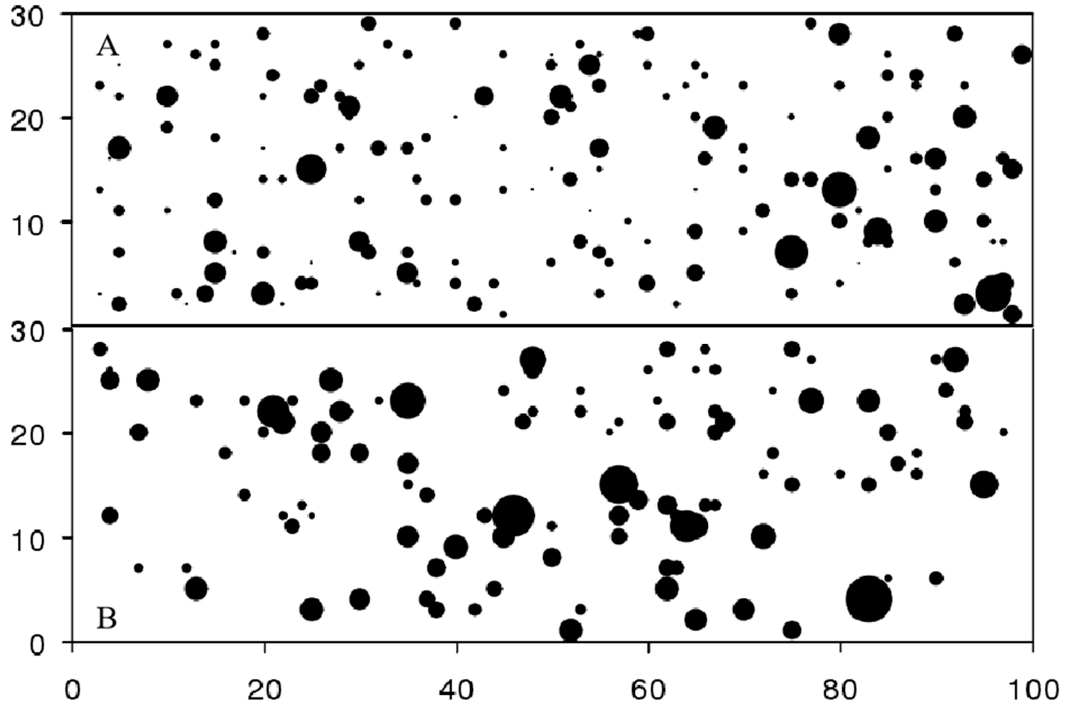

Figure S1 Location and area of anthills in the light degradation sites (A) and the severe degradation sites (B). The area of both light degradation sites and severe degradation sites is 30 m × 100 m. The black spots represent anthills and are presented as 20 times the size of the actual anthills.

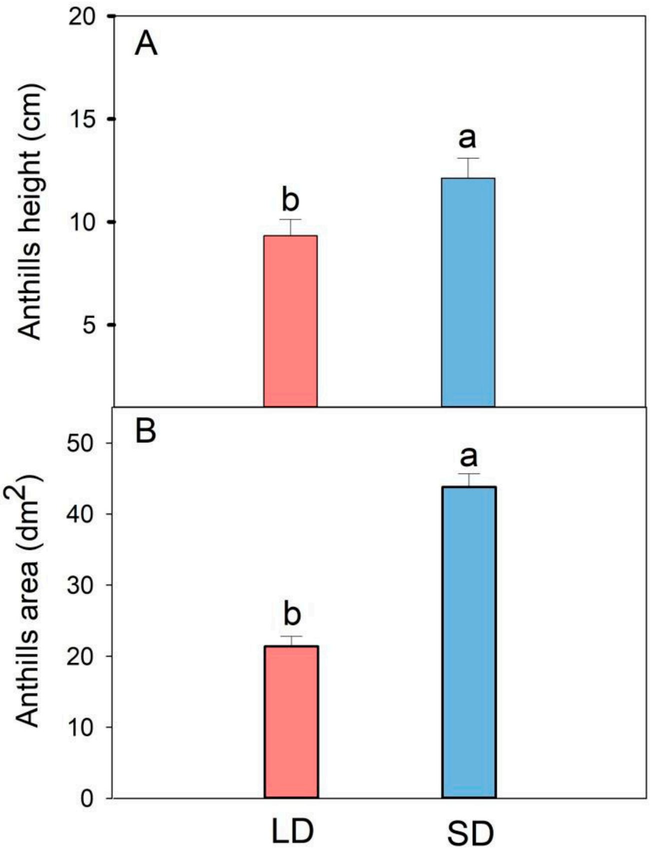

Figure S2 Effects of degradation on the anthills height (A) and area (B). LD: the light degradation sites; SD: the severe degradation sites. Different lowercase letters indicate significant difference between LD and SD, $p < 0.05$. Values = means ± SE.

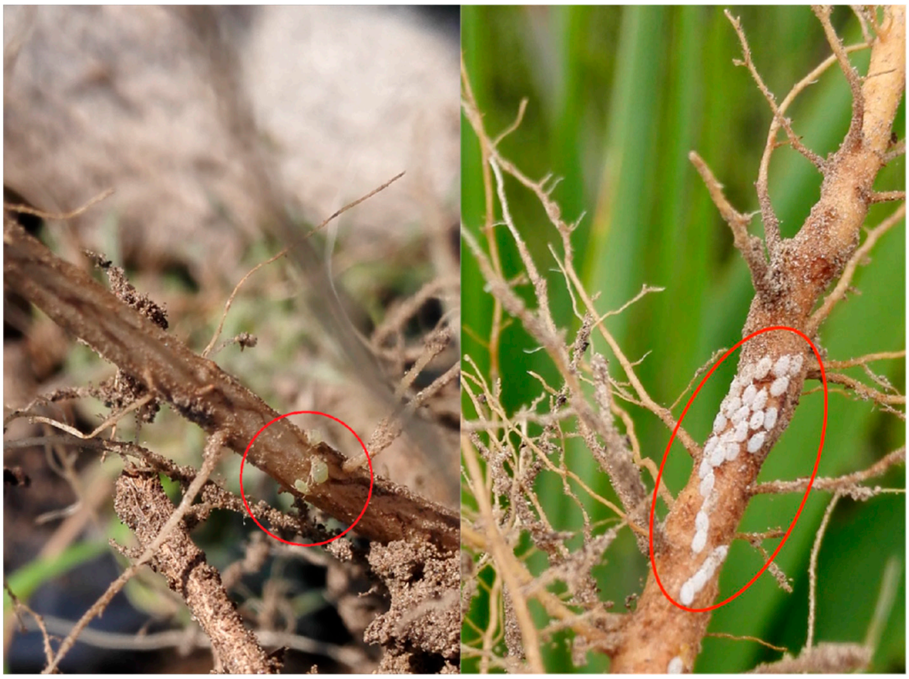

Figure S3 Aphids (*Schizaphis graminum*) and mealybugs (*Pseudococcus comstocki*) on the plant roots.

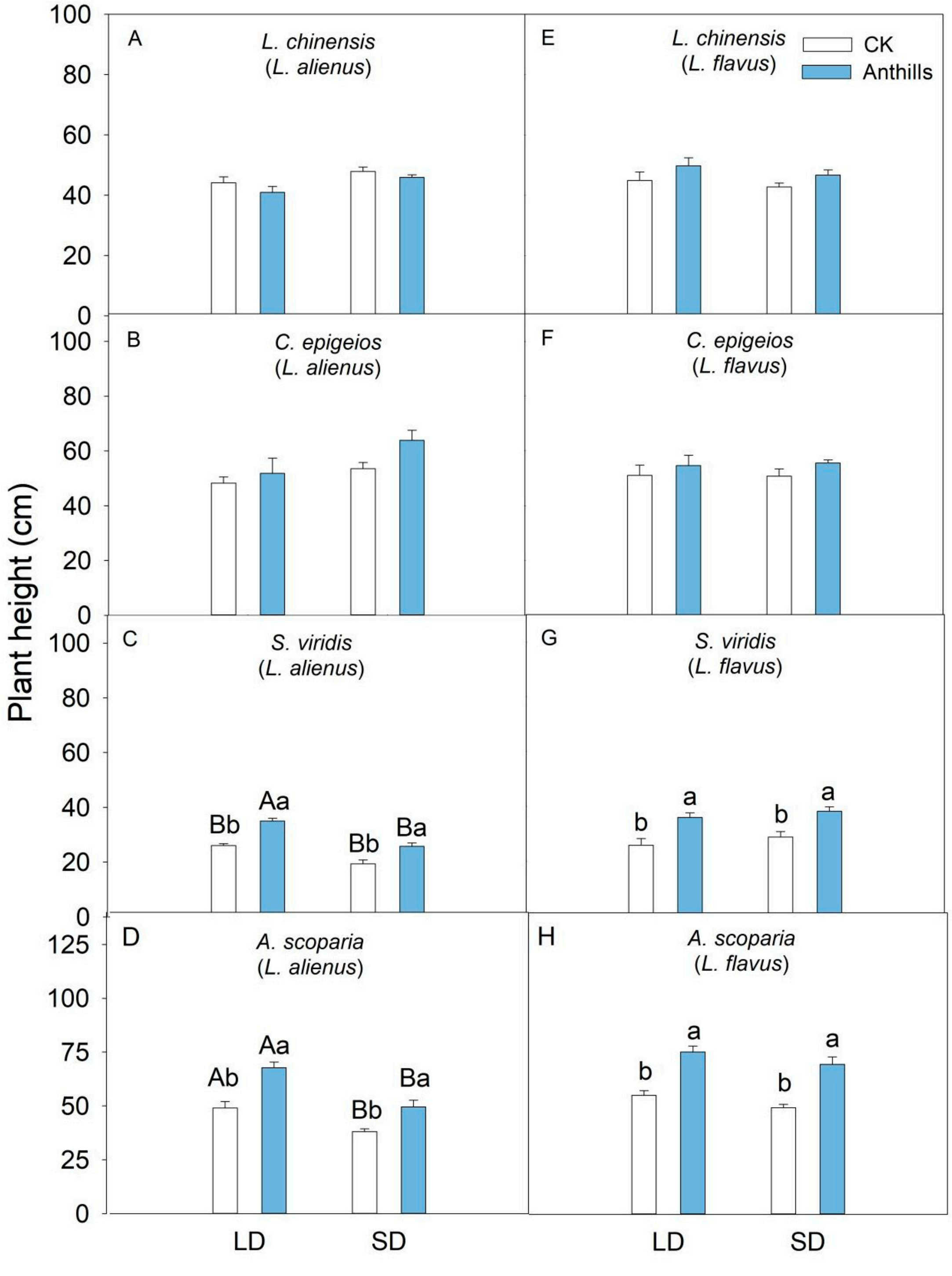

Figure S4 Effects of degradation and anthills on the height of different plant species. (A), (B), (C) and (D): *L. chinensis*, *C. epigeios*, *S. viridis* and *A. scoparia* on the *L. alienus* anthills; (E) , (F), (G) and (H): *L. chinensis*, *C. epigeios*, *S. viridis* and *A. scoparia* on the *L. flavus* anthills. LD: the light degradation sites; SD: the severe degradation sites. Different lowercase letters indicate significant difference between anthills and surrounding vegetation (CK), $p < 0.05$; different capital letters indicate significant difference between LD and SD, $p < 0.05$. Values = means ± SE.

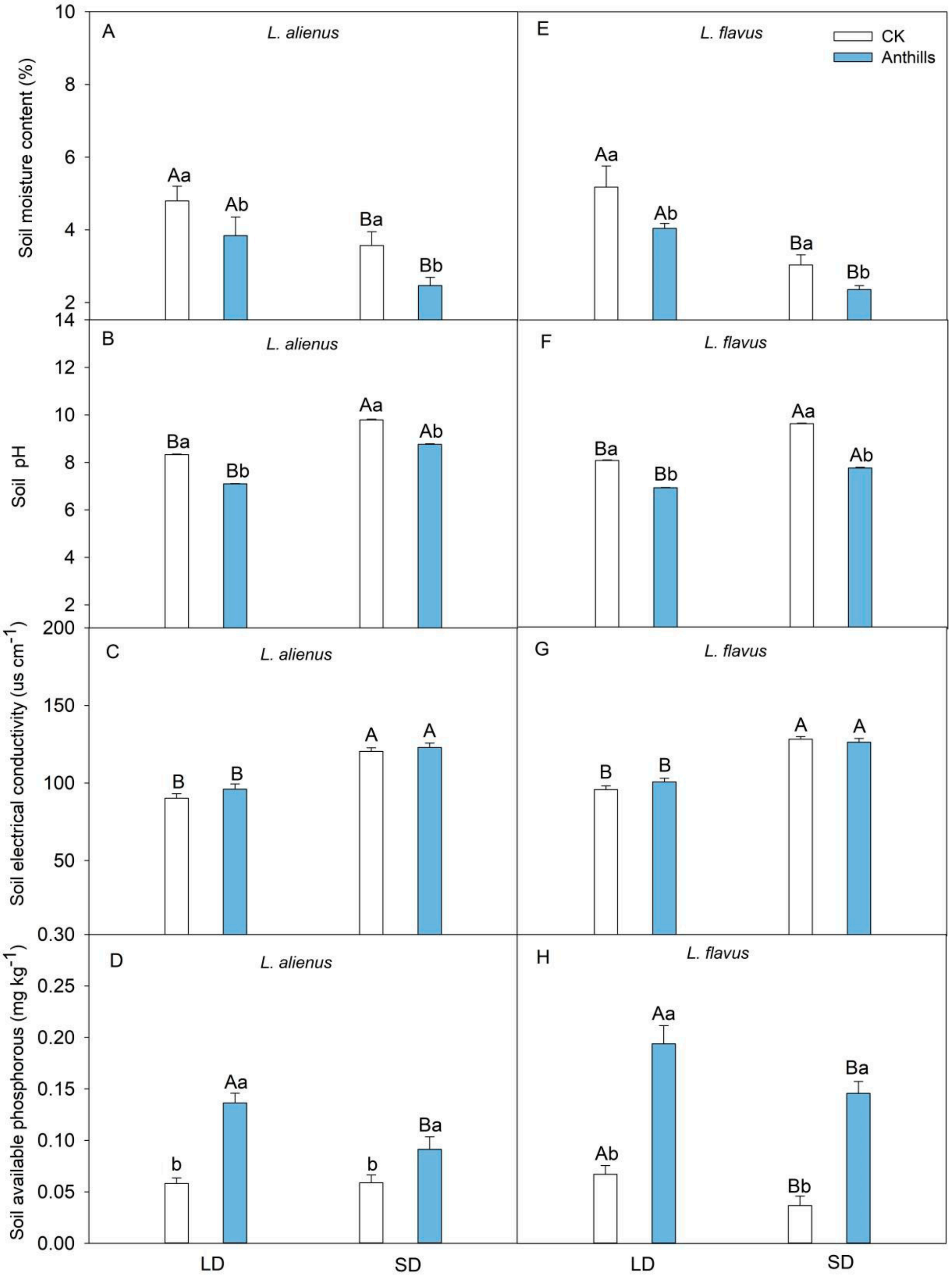

Figure S5 Effects of degradation and anthills on soil properties. Soil moisture content of *L. alienus* anthills (A) and *L. flavus* anthills (E); soil pH value of *L. alienus* anthills (B) and *L. flavus* anthills (F); soil electrical conductivity of *L. alienus* anthills (C) and *L. flavus* anthills (G); soil available phosphorous of *L. alienus* anthills (D) and *L. flavus* anthills (H). LD: the light degradation sites; SD: the severe degradation sites. Different lowercase letters indicate significant difference between anthills and surrounding vegetation (CK), $p < 0.05$; different capital letters indicate significant difference between LD and SD, $p < 0.05$. Values = means ± SE.

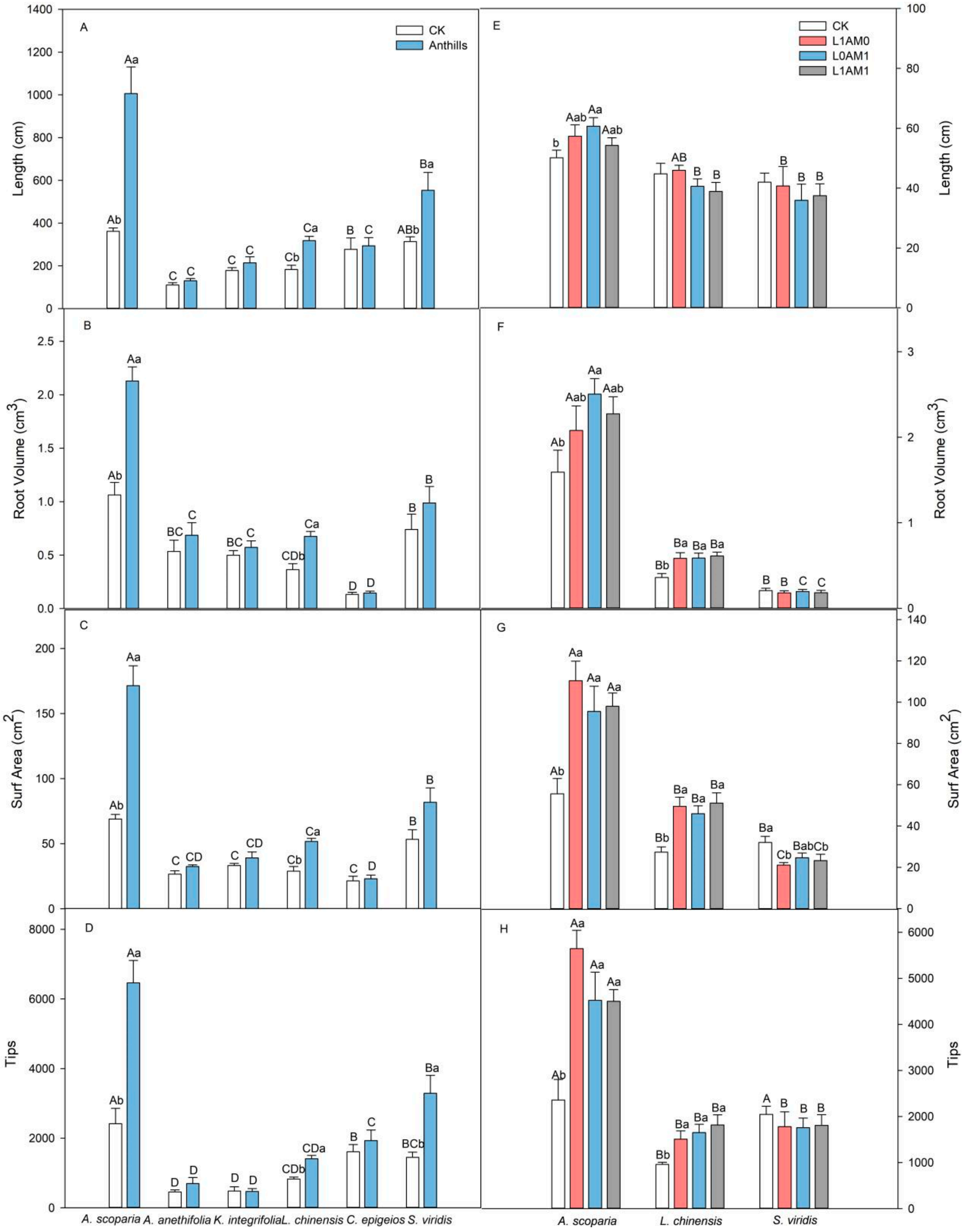

Figure S6 The root morphology including length (A), volume (B), surface area (C) and tips (D) of different plants in the field. The root morphology including length (F), volume (F), surface area (G) and tips (H) of different plants in the greenhouse. Different capital letters indicate significant difference among different plants, *p* < 0.05; different lowercase letters indicate significant difference among different treatments, *p* < 0.05. Values = means ± S.

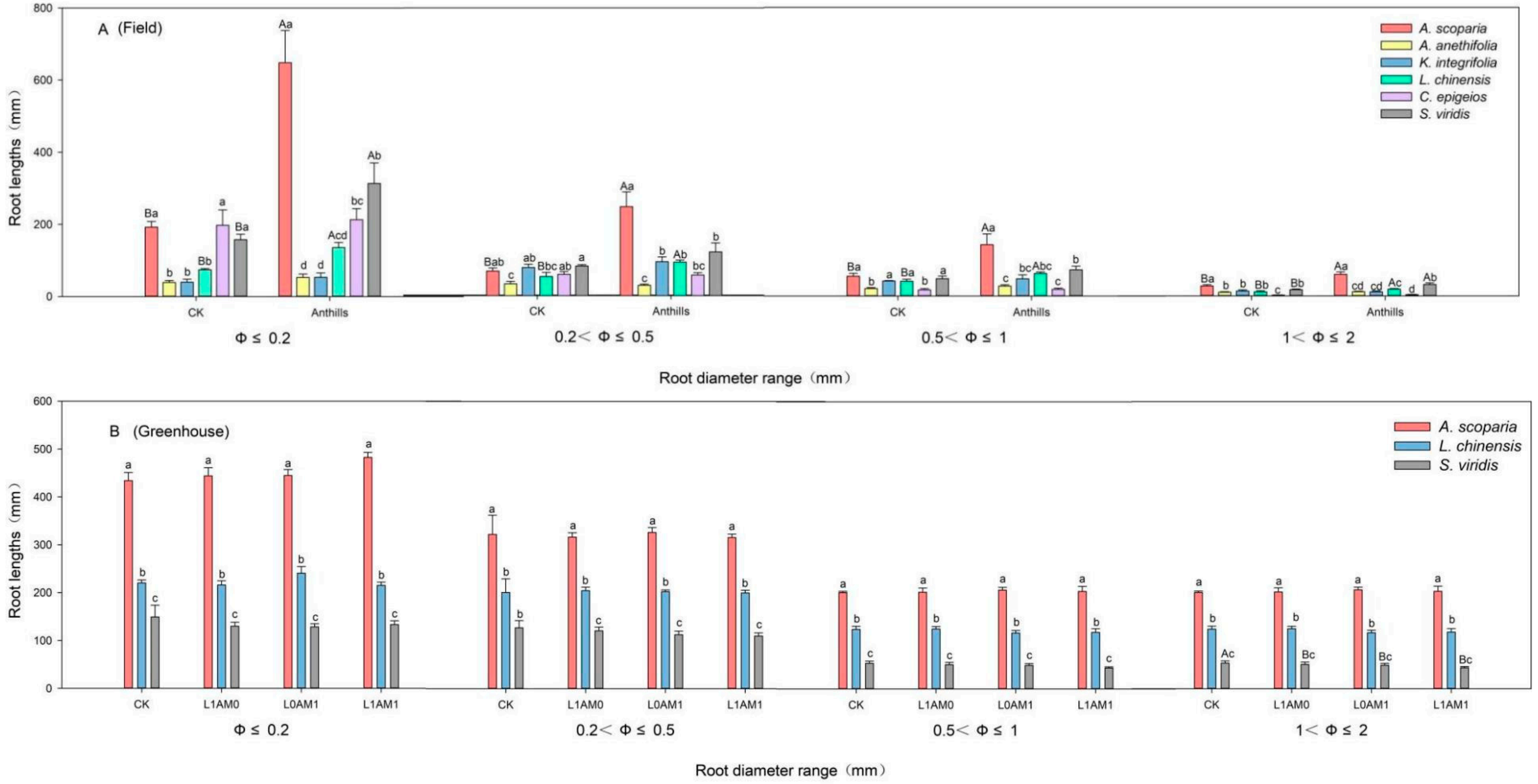

Figure S7 The root length of different root diameters of different plants in the field (A) and greenhouse (B). Different lowercase letters indicate significant difference of root length among different plant species at identical root diameter range, $p < 0.05$; different capital letters indicate significant difference among different treatments, $p < 0.05$. Values = means ± SE.

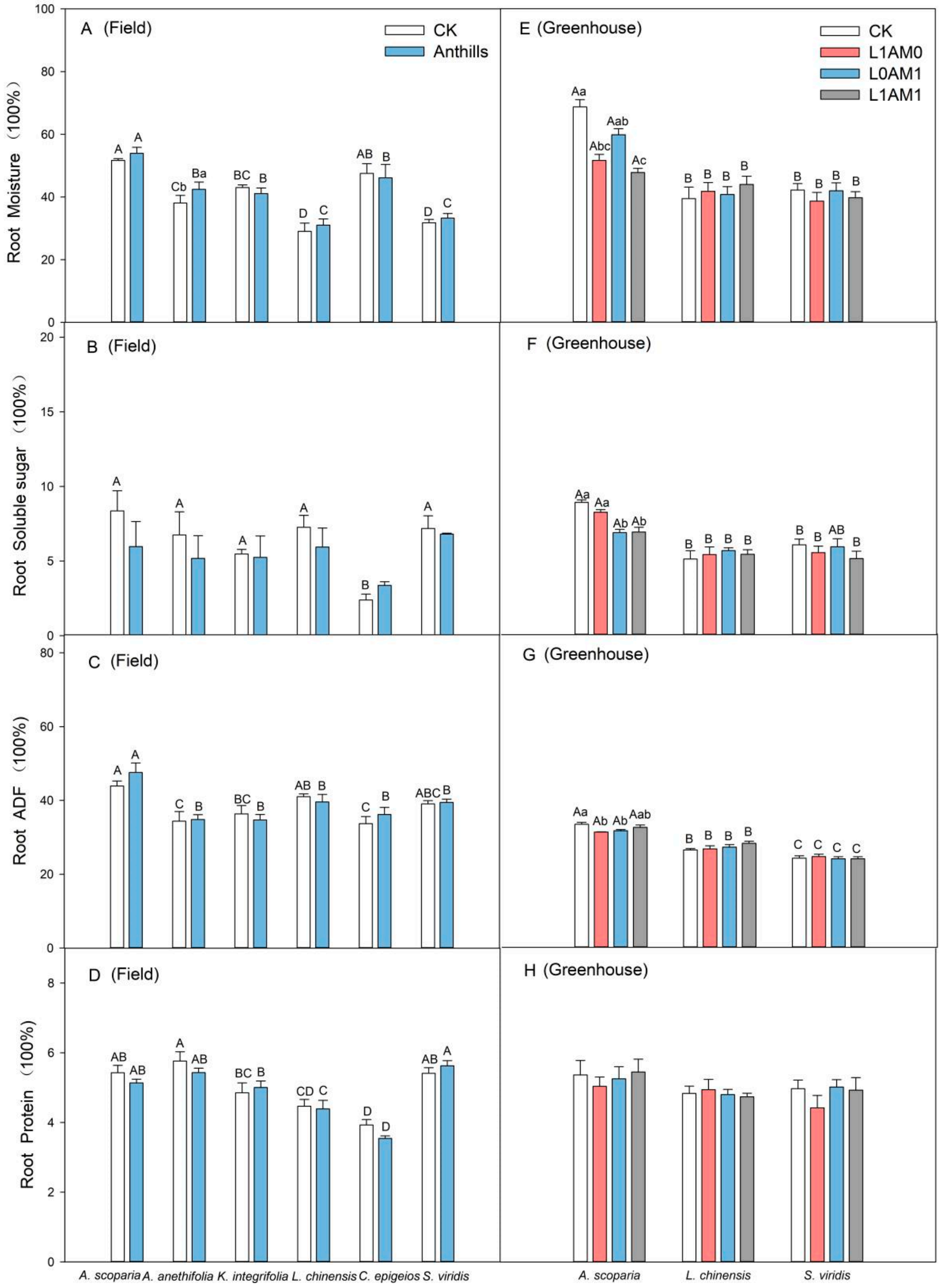

Figure S8 The root nutrition including root moisture content (A), root soluble sugar (B), root ADF (C) and root crude protein (D) of different plants in the field. The root nutrition including root moisture content (E), root soluble sugar (F), ADF (G) and root protein (H) of different plants in the greenhouse. Different capital letters indicate significant difference among different plants, $p < 0.05$; different lowercase letters indicate significant difference among different treatments, $p < 0.05$. Values = means ± SE.

Table S1 Grassland degradation indicator in the light and severe degradation sites. LD: the light degradation sites; SD: the severe degradation sites.

Grassland degradation indicator	Grassland degradation level	
	LD	SD
Alkali spot area	18.5%	69.1%
Vegetation coverage	81.5%	30.9%
Above-ground biomass (g/m^2)	588.8	520.6

Table S2 Description of plant community composition in the experimental sites. LD: the light degradation sites; SD: the severe degradation sites. The symbols + or – indicates presence or absence of this plant species.

Plant species	Function groups	Grassland degradation level	
		LD	SD
Leymus chinensis	Poaceae	+	+
Calamagrostis epigeios		+	+
Phragmites australis		+	+
Setaria viridis		+	+
Chloris virigata		+	+
Arundinella hirta		-	+
Messerschmidia sibirica	Forbs	+	+
Cynanchum chinense		+	+
Equisetum arvense		+	+
Astragalus adsurgens		+	+
Scirpus validus		+	+
Taraxacum mongolicum		+	-
Carex duriuscula		+	+
Lespedeza bicolor		+	+
Potentilla flagellaris		+	+
Saussurea japonica		+	+
Allium neriniflorum		-	+
Kochia sieversiana		-	+
Thalictrum squarrosum		-	+
Medicago sativa		-	+
Inula japonica		-	+
Allium ramosum		-	+
Artemisia scoparia	Asteraceae	+	+
Artemisia anethifolia		+	+
Artemisia mongolica		+	+
Kalimeris integrifolia		+	+

Table S3 Description of plant species abundance in the experimental sites.

Plant species	Abundance	Plant species	Abundance
Setaria viridis	5434	*Artemisia mongolica*	41
Leymus chinensis	4351	*Potentilla flagellaris*	40
Calamagrostis epigeios	1623	*Lespedeza bicolor*	32
Artemisia scoparia	833	*Arundinella hirta*	27
Kalimeris integrifolia	611	*Scirpus validus*	21
Artemisia anethifolia	393	*Medicago sativa*	21
Phragmites australis	371	*Cynanchum chinense*	10
Carex duriuscula	357	*Thalictrum squarrosum*	8
Chloris virigata	121	*Saussurea japonica*	7
Equisetum arvense	103	*Kochia sieversiana*	7
Astragalus adsurgens	89	*Allium ramosum*	5
Messerschmidia sibirica	74	*Taraxacum mongolicum*	4
Allium neriniflorum	57	*Inula japonica*	1

Table S4 Species composition and abundance of seed bank in the soil of anthills and surrounding (CK). LD: the light degradation sites; SD: the severe degradation sites. Different lowercase letters indicate significant difference between anthills and CK soil, $P < 0.05$.

Species	LD			SD		
	L. alienus	*L. flavus*	CK	*L. alienus*	*L. flavus*	CK
Setaria viridis	470±240^{a}	430±128^{a}	290±67^{b}	710±80^{a}	570±76^{a}	60±31^{b}
Artemisia scoparia	330±93^{b}	610±96^{a}	280±72^{b}	420±84^{b}	700±92^{a}	380±106^{b}
Kalimeris integrifolia	30±22^{a}	0	0	30±20^{a}	0	0
Leymus chinensis	100±47^{a}	80±53^{a}	20±20^{b}	14±10^{b}	10±10^{b}	50±30^{a}
Chenopodium glaucum	40±26^{a}	0	0	0	0	0
Persicaria chinensis	20±20^{a}	0	0	0	0	0
Artemisia anethifolia	0	60±34^{a}	10±10^{a}	20±20^{a}	70±36^{a}	30±30^{a}
Echinochloa crusgalli	0	20±20^{a}	0	10±10^{a}	30±21^{a}	0
Polygonum sibiricum	0	0	0	0	20±20^{a}	10±10^{a}
Ixeris polycephala	0	20±20^{a}	0	0	0	10±10^{a}
Inula japonica	0	0	10±10^{a}	0	40±27^{a}	0
Puccinellia tenuiflora	0	0	0	20±20^{a}	0	0
Messerschmidia sibirica	0	10±10^{a}	0	0	20±20^{a}	10±10^{a}
Total	990±190^{ab}	1230±154^{a}	610±89^{b}	1350±124^{a}	1460±238^{a}	550±196^{b}

Table S5 Two-way ANOVA for the effect of degradation, anthills and interaction on the plant abundance of *L. chinensis*, *C. epigeios*, *A. scoparia* and *S. viridis* on the *L. alienus* and *L. flavus* anthills.

Species	Factors	*S. viridis*		*L. chinensis*		*C. epigeios*		*A. scoparia*	
		F	***P***	*F*	***P***	*F*	***P***	*F*	***P***
L. alienus	Degradation	12.731	**0.003****	0.488	**0.489**	0.456	**0.504**	11.266	**0.002****
	Anthills	18.335	**<0.001*****	4.416	**0.043***	0.001	**0.976**	1.286	**0.264**
	Degradation × Anthills	0.004	**0.951**	0.916	**0.345**	0.004	**0.952**	0.249	**0.621**
L. flavus	Degradation	0.307	**0.583**	0.525	**0.473**	1.442	**0.238**	5.018	**0.003****
	Anthills	0.023	**0.879**	3.748	**0.041***	0.7	**0.408**	23.25	**<0.001*****
	Degradation × Anthills	0.154	**0.697**	2.313	**0.137**	0.022	**0.882**	0.144	**0.706**

Table S6 Two-way ANOVA for the effect of plant species, treatment and interaction on the abundance of aphid/mealybugs in the field, the abundance of aphid/mealybugs and ants in the greenhouse.

	Factors	Field		Greenhouse	
		F	***P***	*F*	***P***
Aphid/mealybugs abundance	Species (S)	14.727	**< 0.001*** **	81.355	**< 0.001*** **
	Treatments (T)	13.791	**0.001** **	9.29	**0.006** **
	S×T	6.001	**< 0.001*** **	7.161	**0.004** **
Ants abundance	Species (S)	**-**	-	17.063	**0.001** **
	Treatments (T)	**-**	-	10.563	**0.003** **
	S×T	**-**	-	8.313	**0.002** **

Table S7 One-way ANOVA for the effect of ants abundance to the abundance of aphid/mealybugs in the root of *A. scoparia*, *L. chinensis and S. viridis*; and the effect of aphid/mealybugs abundance to the abundance of ants in the root of *A. scoparia*, *L. chinensis and S. viridis*.

	Factors	*A. scoparia*		*L. chinensis*		*S. viridis*	
Aphids/mealy-bugs abundance	Ants abundance	*F*	***P***	*F*	***P***	*F*	***P***
		28.8	**0.001****	0	**1**	0.167	**0.694**
Ants abundance	Aphids/mealy-bugs abundance	*A. scoparia*		*L. chinensis*		*S. viridis*	
		F	***P***	*F*	***P***	*F*	***P***
		16.133	**0.004****	0	**1**	0.286	**0.608**

Table S8 Two-way ANOVA for the effect of degradation, anthills and interaction on the plant height of *L. chinensis*, *C. epigeios*, *A. scoparia* and *S. viridis* on the *L. alienus* and *L. flavus* anthills.

Species	Factors	*L. chinensis*		*C. epigeios*		*A. scoparia*		*S. viridis*	
		F	***P***	*F*	***P***	*F*	***P***	*F*	***P***
L. alienus	Degradation	4.650	**0.057**	5.400	**0.054**	25.590	**<0.001*** **	49.430	**<0.001*** **
	Anthills	1.980	**0.178**	3.520	**0.079**	27.400	**<0.001*** **	45.380	**<0.001*** **
	Degradation × Anthills	1.350	**0.262**	0.860	**0.368**	3.850	**0.067**	1.350	**0.263**
L. flavus	Degradation	0.050	**0.829**	0.010	**0.909**	18.170	**0.138**	1.850	**0.063**
	Anthills	0.550	**0.467**	1.890	**0.186**	44.460	**<0.001*** **	26.030	**<0.001*** **
	Degradation × Anthills	0.100	**0.759**	0.040	**0.836**	0.480	**0.499**	0.050	**0.834**

Table S9 Two-way ANOVA for the effect of degradation, anthills and interaction on the soil moisture content, soil pH, soil electrical conductivity and soil available phosphorous of the *L. alienus* and *L. flavus* anthills soil.

Species	Factors	Soil moisture content		Soil pH		Soil electrical conductivity		Soil available phosphorous	
		F	***P***	*F*	***P***	*F*	***P***	*F*	***P***
L. alienus	Degradation	10.933	**0.002****	3901.915	**<0.001 *****	98.439	**<0.001 *****	6.481	**0.015***
	Anthills	6.839	**0.013***	2046.194	**<0.001 *****	2.080	**0.158**	69.863	**<0.001 *****
	Degradation× Anthills	0.036	**0.850**	17.453	**<0.001 *****	0.329	**0.570**	6.092	**0.018***
L. flavus	Degradation	32.752	**<0.001 *****	2435.185	**<0.001 *****	168.378	**<0.001 *****	10.546	**0.003****
	Anthills	7.338	**0.010***	3911.270	**<0.001 *****	0.422	**0.520**	31.481	**<0.001 *****
	Degradation× Anthills	0.473	**0.496**	217.512	**<0.001 *****	2.391	**0.131**	0.541	**0.467**

Table S10 Two-way ANOVA for the effect of plant species, treatments and interaction on the root morphology (root length, volume, length/volume, project area, surface area, tips, length of different root diameter).

	Factors	Field		Greenhouse	
		F	***P***	*F*	***P***
Root Length (cm)	Species (S)	33.538	**< 0.001*****	22.565	**< 0.001*****
	Treatments (T)	40.298	**< 0.001*****	0.733	**0.537**
	S×T	11.977	**< 0.001*****	1.088	**0.383**
Root Volume (cm^3)	Species (S)	52.163	**< 0.001*****	213.446	**< 0.001*****
	Treatments (T)	31.16	**< 0.001*****	4.043	**0.012***
	S×T	8.012	**< 0.001*****	2.16	**0.063**
Root Proj Area (cm^2)	Species (S)	52.376	**< 0.001*****	173.782	**< 0.001*****
	Treatments (T)	46.417	**< 0.001*****	13.098	**< 0.001*****
	S×T	15.319	**< 0.001*****	10.453	**< 0.001*****
Root Surf Area (cm^2)	Species (S)	65.373	**< 0.001*****	125.181	**< 0.001*****
	Treatments (T)	57.177	**< 0.001*****	8.303	**< 0.001*****
	S×T	17.718	**< 0.001*****	5.829	**< 0.001*****
Root Tips	Species (S)	47.988	**< 0.001*****	94.97	**< 0.001*****
	Treatments (T)	44.722	**< 0.001*****	8.308	**< 0.001*****
	S×T	13.11	**< 0.001*****	6.52	**< 0.001*****
Root Length（mm）（Φ≤0.2）	Species (S)	34.307	**< 0.001*****	649.856	**< 0.001*****
	Treatments (T)	32.913	**< 0.001*****	0.614	**0.609**
	S×T	12.08	**< 0.001*****	1.788	**0.122**
Root Length（mm）(0.2＜Φ≤0.5)	Species (S)	24.455	**< 0.001*****	157.811	**< 0.001*****
	Treatments (T)	15.209	**< 0.001*****	0.136	**0.938**
	S×T	9.513	**< 0.001*****	0.104	**0.996**
Root Length（mm）(0.5＜Φ≤1)	Species (S)	16.282	**< 0.001*****	646.921	**< 0.001*****
	Treatments (T)	14.992	**< 0.001*****	0.346	**0.792**
	S×T	4.548	**0.002****	0.384	**0.885**
Root Length（mm）(1＜Φ≤2)	Species (S)	26.617	**< 0.001*****	690.011	**< 0.001*****
	Treatments (T)	43.103	**< 0.001*****	4.346	**0.009****
	S×T	8.728	**< 0.001*****	1.106	**0.373**

Table S11 Two-way ANOVA for the effect of plant species, treatments and interaction on the root nutrition (root moisture content, root soluble sugar, root ADF, root crude protein).

	Factors	Field		Greenhouse	
		F	***P***	*F*	***P***
Root moisture content	Species (S)	28.537	**< 0.001*****	2.866	**0.046***
	Treatments (T)	0.73	**0.401**	35.492	**< 0.001*****
	S×T	0.555	**0.733**	3.43	**0.007****
Root soluble sugar	Species (S)	4.13	**0.008****	2.712	**0.055**
	Treatments (T)	1.643	**0.212**	43.949	**< 0.001*****
	S×T	0.578	**0.717**	2.822	**0.02***
Root ADF	Species (S)	11.773	**< 0.001*****	1.043	**0.382**
	Treatments (T)	0.411	**0.528**	202.39	**< 0.001*****
	S×T	0.697	**0.631**	1.924	**0.096**
Root crude protein	Species (S)	28.859	**< 0.001*****	0.511	**0.676**
	Treatments (T)	1.176	**0.289**	3.046	**0.057**
	S×T	0.924	**0.483**	0.42	**0.862**
